# At the Crossroads of the Adipocyte and Osteoclast Differentiation Programs: Future Therapeutic Perspectives

**DOI:** 10.3390/ijms21072277

**Published:** 2020-03-26

**Authors:** Shanmugam Muruganandan, Andreia M. Ionescu, Christopher J. Sinal

**Affiliations:** 1 Department of Developmental Biology, Harvard School of Dental Medicine, 188 Longwood Avenue, Boston, MA 02115, USA; andreia_ionescu@hms.harvard.edu; 2 Department of Pharmacology, Dalhousie University, Halifax, NS B3H 4R2, Canada; christopher.sinal@dal.ca

**Keywords:** adipocyte osteoclast cross talk ppar gamma, cebp alpha, cebp beta

## Abstract

The coordinated development and function of bone-forming (osteoblasts) and bone-resorbing (osteoclasts) cells is critical for the maintenance of skeletal integrity and calcium homeostasis. An enhanced adipogenic versus osteogenic potential of bone marrow mesenchymal stem cells (MSCs) has been linked to bone loss associated with diseases such as diabetes mellitus, as well as aging and postmenopause. In addition to an inherent decrease in bone formation due to reduced osteoblast numbers, recent experimental evidence indicates that an increase in bone marrow adipocytes contributes to a disproportionate increase in osteoclast formation. Therefore, a potential strategy for therapeutic intervention in chronic bone loss disorders such as osteoporosis is to interfere with the pro-osteoclastogenic influence of marrow adipocytes. However, application of this approach is limited by the extremely complex regulatory processes in the osteoclastogenic program. For example, key regulators of osteoclastogenesis such as the receptor activator of nuclear factor-kappaB ligand (RANKL) and the soluble decoy receptor osteoprotegerin (OPG) are not only secreted by both osteoblasts and adipocytes, but are also regulated through several cytokines produced by these cell types. In this context, biologically active signaling molecules secreted from bone marrow adipocytes, such as chemerin, adiponectin, leptin, visfatin and resistin, can have a profound influence on the osteoclast differentiation program of hematopoietic stem cells (HSCs), and thus, hold therapeutic potential under disease conditions. In addition to these paracrine signals, adipogenic transcription factors including CCAAT/enhancer binding protein alpha (C/EBPα), C/EBP beta (C/EBPβ) and peroxisome proliferator-associated receptor gamma (PPARγ) are also expressed by osteoclastogenic cells. However, in contrast to MSCs, activation of these adipogenic transcription factors in HSCs promotes the differentiation of osteoclast precursors into mature osteoclasts. Herein, we discuss the molecular mechanisms that link adipogenic signaling molecules and transcription factors to the osteoclast differentiation program and highlight therapeutic strategies targeting these mechanisms for promoting bone homeostasis.

## 1. Introduction

Bone is a dynamic connective tissue that continuously undergoes homeostatic remodeling throughout life via a tightly regulated balance between the resorption of old bone and the formation of new bone tissue [1,2,3,4,5,6,7]. Osteoblasts are the primary bone formative cell type while osteoclasts are the key resorptive cells that govern bone remodeling and maintain skeletal integrity [1,2,4,5,6]. A tight coupling between the formation and function of osteoblasts and osteoclasts is required to maintain the homeostatic balance between bone formation and resorption. Two distinct self-renewing populations of multipotent stem cells reside within bone marrow—mesenchymal stem cells (MSCs) that give rise to the mesenchymal lineages, including osteoblasts and hematopoietic stem cells (HSCs) that give rise to all blood cell types including the monocyte lineage, from which osteoclasts are derived [3,6,8]. Under normal conditions, coordinated bone remodeling is achieved through a complex paracrine signaling network that regulates the osteoblastogenic and osteoclastogenic programs of the respective stem cell precursors [1,2,3,4,5,6,7,8]. For example, receptor activator of nuclear factor-kappa B ligand (RANKL) is an indispensable factor for osteoclast differentiation of hematopoietic lineage cells, but is primarily produced by cells of the osteoblast lineage [9,10]. Furthermore, among the isoforms of RANKL produced in bone marrow, the membrane-bound form, which requires cell-to-cell contact between osteoblast precursors and HSCs for activity, is more effective than the secreted soluble forms at promoting osteoclastogenesis [11,12]. Similarly, initiation of the osteoblast developmental program is largely dependent upon signals from cells of the osteoclast lineage within the bone microenvironment [13,14,15,16,17]. For instance, retrograde signaling transduced by osteoclast precursors through surface-bound RANKL molecules on osteoblast precursors induces an osteoblastogenic stimulus in the osteoblast precursors [13,14,15]. Thus, the current literature provides experimental evidence for the existence of both osteoblast-mediated osteoclastogenic and osteoclast-mediated osteoblastogenic differentiation through the bidirectional RANKL-RANK signaling system that serves to coordinate the local development and activities of osteoblast and osteoclast precursor cells. 

In addition to osteoblasts, bone marrow MSCs are also able to differentiate into adipocytes. It is well-established that the adipogenic and osteoblastogenic differentiation programs are competitively balanced such that mechanisms which promote adipogenesis actively suppress the osteoblastogenic program [3,6,18]. Several osteoporotic bone loss disorders associated with age, postmenopause and chronic diseases such as diabetes mellitus are characterized by an increase in bone marrow adipogenesis and reduced bone formation [3,6,18]. Consistent with the competitive nature of lineage allocation, adipogenic transcription factors including peroxisome proliferator-associated receptor gamma (PPARγ) and CCAAT/enhancer binding protein alpha (C/EBPα) have been shown to suppress the MSC osteoblastogenic program [3,19,20,21,22]. The activities of these adipogenic transcription factors, as well as osteoblastogenic signaling pathways such as Wnt/β-catenin are influenced by extracellular factors present within the local bone microenvironment. For example, our previous studies provide evidence for a negative regulation of the osteoblastogenic program by the adipocyte secreted signaling molecule (adipokine) chemerin. Chemerin activates the cognate receptor chemokine-like receptor 1 (CMKLR1) to suppress the bone-anabolic Wnt/β-catenin and Notch signaling pathways in MSCs [23]. However, other adipokines such as leptin, adiponectin and omentin-1 have been shown to stimulate osteoblast differentiation [6,24,25,26]. Although mixed results have been observed for adipokines, the majority of commonly occurring bone loss disorders are associated with increased numbers of bone marrow adipocytes [18]. Thus, increased bone marrow adipogenesis can adversely affect bone remodeling via a direct negative impact upon the MSC osteoblast differentiation program. 

Recent studies have revealed that adipogenic transcription factors including PPARγ, C/EBPα and CEBPβ are also expressed and activated in hematopoietic lineage cells during osteoclast differentiation and are critical for the lineage priming, differentiation and activity of osteoclasts [27,28,29,30,31,32,33,34,35]. Furthermore, several adipokines are reported to influence the osteoclastogenic program of HSCs suggesting that increased bone marrow adipogenesis could exert further detrimental effects on bone health by uncoupling osteoblast-osteoclast communication in a fashion that promotes osteoclastogenesis and bone resorption [6,7,36,37,38]. In the present review, we discuss recent progress in the understanding of the influence of adipogenic events on the osteoclast differentiation program and highlight the future therapeutic potential for targeting these pathways to promote bone regeneration in disorders of bone loss.

## 2. Regulation of Osteoclastogenesis Through the Adipogenic Program

Until recently, studies on osteoclast recruitment, differentiation and function have focused primarily on osteoblast lineage-derived molecules such as macrophage-colony stimulating factor (MCSF), RANKL and osteoprotegerin (OPG) [9,10,39]. The intracellular osteoclastogenic pathways linked to these stimuli are mediated through distinct RANK-mediated activation of tumor necrosis factor receptor-associated factors (TRAFs) that leads to the feed forward expression and activation of nuclear factor-kappa B (NFκB), c-fos and nuclear factor of activated T cells 1 (NFATc1) in HSCs, ultimately inducing the osteoblast-dependent osteoclast differentiation [40,41,42]. However, several recent studies have addressed the intracellular mechanisms that are interwoven between the adipogenic and osteoclastogenic programs [27,28,29,30,31,32]. In this context, it has been reported that some of the key players known to regulate MSC adipocyte differentiation are expressed and crucial for HSC osteoclast differentiation and likewise, the major osteoclast differentiation factor RANKL is highly expressed in bone marrow adipocytes [27,28,29,30,31,32,33,34,43,44,45,46]. These findings have recently gained greater attention due to their disease relevance since the control of osteoclast differentiation under these conditions could be driven by an adipogenic program, regardless of the status of the osteoblastogenic differentiation program [46]. Conceptually, this was first demonstrated directly by studies showing that bone marrow adipocytes can induce HSC osteoclastogenesis even in the absence of osteogenic lineage cells [44,45]. Thus, the involvement of key adipogenic transcription factors such as C/EBPα, C/EBPβ and PPARγ in driving HSC osteoclast differentiation, coupled with the expression of RANKL by marrow adipocytes, provide an efficacious stimulus that can uncouple osteoclastogenesis from paracrine signaling by preosteoblasts under disease conditions. Moreover, other bone marrow adipocyte-secreted molecules have also been shown to interact with their cognate receptors on HSCs to promote osteoclast differentiation. Together, these studies suggest that bone marrow adipocytes are not only a positive regulator of HSC osteoclastogenesis, but developmentally, HSCs also share an intertwined signaling system with the adipogenic program that is amenable to activation by diseases promoting bone marrow adipogenesis.

## 3. Transcription Factors in the Intertwining of Adipogenesis and Osteoclastogenesis

### 3.1. PPARγ: The Master Adipogenic Transcription Factor

A key finding in the field of osteoclast development was that among all the hematopoietic stem cell and progenitor populations in bone marrow, those that express PPARγ are specifically endowed with the ability to commit into the osteoclast lineage [29]. Using in vivo lineage tracing strategies with PPARγ-tTA TRE-H2BGFP reporter mice, Wei et al. (2011) demonstrated that osteoclasts are derived from PPARγ-expressing cells of the hematopoietic lineage [29]. The GFP+ PPARγ+ but not the GFP- PPARγ- bone marrow cells derived from the PPARγ-tTA TRE-H2BGFP reporter mice differentiated into mature osteoclasts when cultured under osteoclastogenic conditions indicating that the osteoclastogenic precursor population lies within the PPARγ+ bone marrow subpopulation. Further, the impact of PPARγ-expressing hematopoietic subsets on bone homeostasis was confirmed by selective depletion of PPARγ+ cells in mice [29]. This was achieved using a novel transgenic mouse model generated by crossing transgenic mice expressing Cre recombinase under the control of the PPARγ promoter driven tetracycline-controlled transactivator protein (tTA) combined with a tetracycline-responsive promoter element (TRE)-Cre transgene (PPARγ-tTA TRE-Cre) with a second line of transgenic mice that exhibited conditional expression of diphtheria toxin (DTA) in the presence of Cre (DTA-floxed). This resulted in the selective ablation of PPARγ^+^ progenitors, ablation of osteoclast differentiation and an osteopetrotic (abnormally dense bone) phenotype in the offspring [29]. Furthermore, specific activation of Notch in the PPARγ^+^ progenitor cell populations by breeding the PPARγ-tTA TRE-Cre mice with a constitutively active Notch Intracellular Domain (NICD)-floxed mice suppressed osteoclast differentiation. This is consistent with a role for Notch activation in the suppression of PPARγ signaling in osteoclastogenic cells [29] that is analogous to the suppression of adipogenesis in MSCs by Notch [23,47,48,49,50]. Taken together, these studies indicate that PPARγ activation can induce adverse effects on skeletal homeostasis through the combined effects of reduced bone formation due to suppression of MSC osteoblastogenesis on one hand, and exacerbated bone resorption due to increased HSC osteoclastogenesis on the other. 

**Figure 1:**
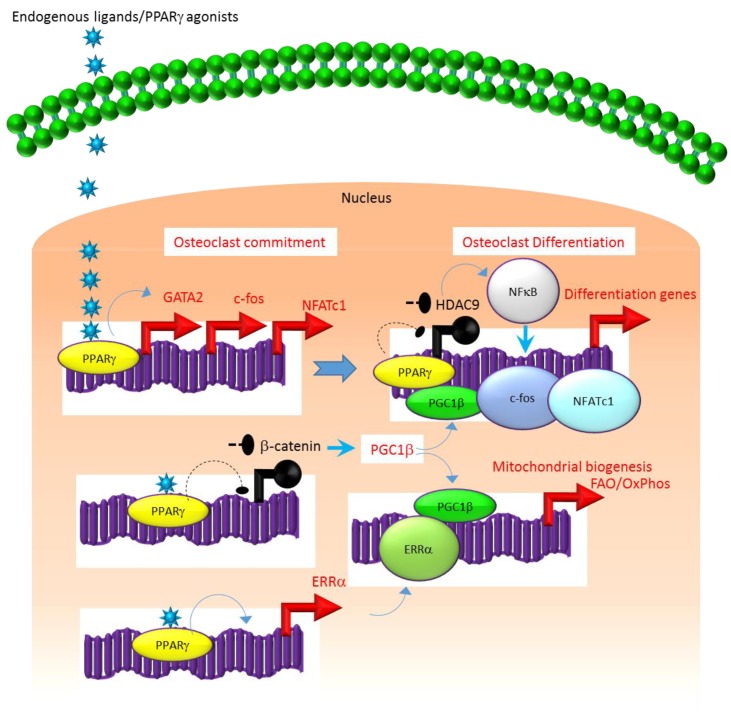
Regulation of osteoclastogenesis by PPARγ. Adipogenic stimulus prevailing in bone marrow can induce osteoclastogenesis by activating PPARγ that in turn activates the transcription of target genes such as GATA2, c-fos, NFATC1 and ERR1α that induce osteoclast differentiation in HSCs. Additionally, suppression of Wnt/β-catenin signaling by PPARγ can induce PGC1β which can function as a transcriptional co-activator for ERR1α to promote the induction of mitochondrial fatty acid β-oxidation and oxidative phosphorylation genes that are critical for inducing mitochondrial biogenesis to support osteoclast function. PGC1β also can serve as a transcriptional co-activator for PPARγ to induce target genes that stimulate osteoclast differentiation. Blue colored arrow-ended lines: stimulation/activation; Black circle-ended dash lines: inhibition. Red colored pointed arrows: transcriptional activation; Black colored closed circles: transcriptional repression.

A similar association exists between the molecular mechanisms that regulate the expression and function of PPARγ. For instance, several studies suggest that the class II histone deacetylase 9 (HDAC9) acts in a negative feedback loop with adipogenic transcription factors such as PPARγ to regulate MSC adipogenesis by repressing PPARγ expression in the undifferentiated state of MSCs, whereas under adipogenic conditions, the stimulation of PPARγ expression transrepresses HDAC9 gene expression to permit adipogenic gene expression and differentiation [23,51,52]. In parallel to a role in MSC adipogenesis, it was recently reported that HDAC9-knockout (−/−) mice exhibit low bone mass due to exacerbated osteoclast formation and bone resorption [48]. This phenotype could be rescued by transplanting bone marrow from wild-type (WT) mice to HDAC9 (−/−) mice, while transfer of bone marrow from HDAC9 (−/−) mice to WT mice induced the bone resorption phenotype in the latter [53]. This supports the view that the interplay between PPARγ and HDAC9 is relevant to both bone marrow osteoblastogenic and osteoclastogenic cell populations. Further, it is well-established that PPARγ and Wnt/β-catenin form a negative feedback loop in MSCs such that PPARγ drives adipogenesis by transrepressing HDAC9, cyclin D1 and Notch expression and signaling that ultimately limits osteoblastogenic Wnt/β-catenin signaling [23,54]. Parallel to MSCs, it has been reported that PPARγ also downregulates Wnt/β-catenin signaling in HSCs, which in turn induces peroxisome proliferator-activated receptor-gamma coactivator-1 beta (PGC1β) expression during osteoclastogenesis (Figure 1). Mechanistically, PGC1β exhibits dual roles in HSCs by transactivating c-fos expression which induces osteoclast differentiation and by stimulating mitochondrial biogenesis which supports osteoclast function during bone resorption (Figure 1). As such, PGC1β serves as a common mediator for both PPARγ stimulation of osteoclastogenesis and the resorptive functions of mature osteoclasts [28]. In addition, PPARγ also directly induces the expression of estrogen receptor-related receptor alpha (ERRα) which coordinates with PGC1β to induce mitochondrial biogenesis (Figure 1) that additionally contributes to the PPARγ-mediated activation of mature osteoclasts [28]. Consistent with this, targeted deletion of PGC1β in osteoclastogenic cells by breeding PGC1β-floxed mice with Tie2cre-transgenic mice resulted in the abrogation of PPARγ-induced bone resorption [27,28]. Thus, it is now clear that PPARγ is also an integral component of the core transcriptional machinery of HSCs (Figure 1) and activation of this transcription factor under adipogenic conditions in bone marrow can induce osteoclast differentiation and increase bone resorption in disease conditions.

### 3.2. C/EBPα

Similar to PPARγ, considerable experimental evidence also supports a role for the adipogenic transcription factor C/EBPα as a hematopoietic transcription factor [55,56] that promotes the osteoclastogenic differentiation program [30,31,32,33]. 

**Figure 2:**
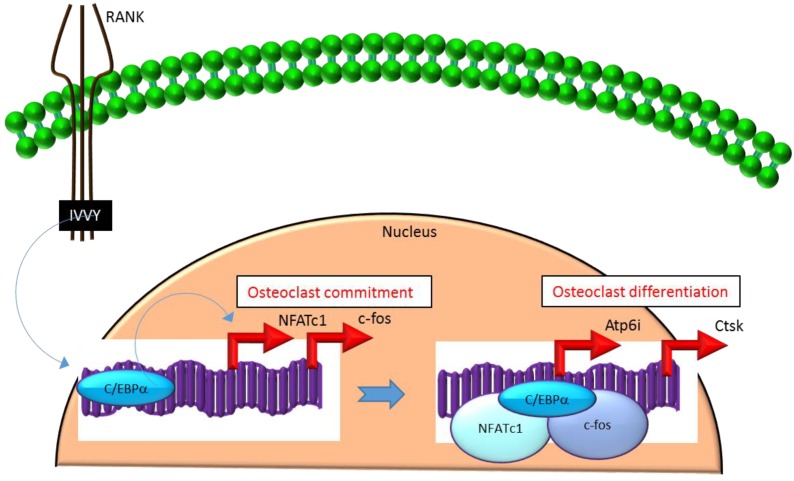
C/EBPα promotes lineage commitment, differentiation and function of osteoclasts. An increase in C/EBPα expression in HSCs is mediated through activation of IVVY motif of the cytoplasmic domain of RANK. C/EBPα can activate transcription of the master osteoclastogenic transcription factor NFATc1 and c-fos to promote osteoclast lineage commitment independent of the presence or absence of RANKL. In addition, C/EBPα can also function as a transcriptional co-activator for NFATc1 and c-fos to induce osteoclastogenic target genes and promote osteoclast differentiation. Blue colored arrow-ended lines: stimulation/activation. Red colored pointed arrows: transcriptional activation.

Mechanistically, C/EBPα serves as an osteoclastogenic factor through its ability to bind DNA and activate the transcription of several osteoclast-specific transcription factors including NFATc1 and c-fos (Figure 2) as well as genes required for osteoclast resorptive function including cathepsin K (Ctsk) and osteoclast-specific vacuolar proton pump (Atp6i, also known as Tcirg1; Figure 2) that promotes osteoclast differentiation and function [31]. Promoter activity mapping and chromatin immunoprecipitation (ChIP) assays have identified cis-regulatory elements (CREs) located in the promoter regions and have identified these as direct regulatory targets of C/EBPα [31] (Figure 2). This has been linked to paracrine osteoclastogenic signaling by the observation that the ^535^IVVY^538^ (IVVY) motif of RANK stimulates C/EBPα expression, which in turn activates the expression of osteoclastogenic genes (Figure 2) and promotes osteoclast differentiation of HSCs [35]. Consistent with this, mutation of the IVVY motif blocked RANKL stimulation of C/EBPα expression and osteoclast differentiation of HSCs [35]. It has been reported that C/EBPα has a substantial and wide-ranging role throughout multiple phases of osteoclast development and function. For example, overexpression of C/EBPα alone was reported to be sufficient to promote osteoclast lineage commitment of HSCs even in the absence of RANKL stimulation [31,35]. Furthermore, ectopic expression of C/EBPα in differentiated macrophages reprogrammed the cells to fuse and convert into osteoclast-like cells with a marked induction of several osteoclast marker genes including TRAP, Ctsk, NFATc1, c-fos, MMP9, TRAF6 and RANK coincident with a downregulation of the macrophage marker F4/80 [31]. Consistent with these findings, C/EBPα^+/−^ and C/EBPα^−/−^ mice were reported to exhibit a gene dosage-dependent loss of osteoclast numbers and an osteopetrosis phenotype [31]. In addition to a role in osteoclast lineage commitment and maturation, C/EBPα is also critical for osteoclast function as it is essential to stimulate the extracellular acidification process and to maintain osteoclast survival during resorption [30]. Together, these studies provide evidence that C/EBPα is an osteoclastogenic transcription factor that can effectively induce osteoclast differentiation and function (Figure 2).

### 3.3. C/EBPβ

C/EBPβ is encoded by an intronless gene that directs the production of four protein isoforms—a 38 kDa full-length C/EBPβ, 34 kDa liver-enriched activating protein (LAP), 21 kDa liver-enriched inhibitory protein (LIP), and a smaller inactive 14-kDa isoform. All of these isoforms are generated by alternative translation initiation from consecutive in-frame start codons directed under the control of the mammalian target of rapamycin kinase (mTOR) pathway [33,34,57,58]. The LIP isoform lacks a major portion of the transactivation domain but retains the DNA-binding domain and dimerization domain that confers its function as a dominant negative regulator of the transactivator isoforms LAP and full-length C/EBPβ [33,34]. It is well-established that regulation of C/EBPβ mRNA expression and the relative levels and turnover of LIP and LAP isoforms play a major role in adipocyte differentiation [59]. For example, constitutive overexpression of C/EBPβ-LIP suppresses adipogenesis, while that of C/EBPβ-LAP promotes adipogenesis by regulating the expression of PPARγ in MSCs [60,61]. The balance between the expression of long transactivator isoforms (full-length or LAP) and the truncated repressor LIP is determined by the mTOR pathway that acts as a key sensor to integrate the differentiation pathways with the nutrient stimuli [33,62,63]. Although some reports suggest that mTOR activates LAP to induce adipogenesis, other evidence indicates that the DEP domain containing the mTOR-interacting protein (DEPTOR) strongly induces PPARγ and adipogenesis by inhibiting mTOR [64]. This suggests that the mechanisms by which C/EBPβ impacts adipocyte differentiation is highly complex and the nature of mTOR signaling determines the relative function of full-length/LAP versus LIP isoforms in inducing or repressing the differentiation program. Similar to the adipogenic cells, the balance between the long transactivator isoforms (full-length/LAP) and the truncated repressor LIP is also regulated by the mTOR pathway in the osteoclastogenic cells [33,34]. Moreover, the osteoclast differentiation program is influenced by the LAP to LIP ratio that determines the activation/repression of C/EBPβ target genes in HSCs [33,34]. In contrast to adipogenesis, HSC osteoclastogenesis is generally suppressed by LAP and stimulated by LIP. 

**Figure 3:**
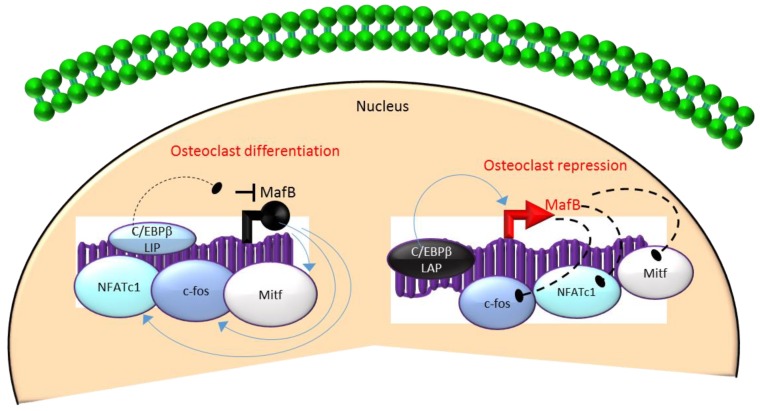
A negative feedback loop between C/EBPβ and MafB regulates osteoclastogenesis. C/EBPβ is produced as two major isoforms: C/EBPβ-LAP (stimulatory) and C/EBPβ-LIP (inhibitory) which activate or repress the basic region/leucine zipper transcription factor MafB, respectively. The repression of MafB by LIP induces osteoclast differentiation due to the loss of MafB-mediated repression of NAFTc1, c-fos and Mitf. In contrast, stimulation of MafB expression by LAP blocks osteoclast differentiation due to MafB-mediated repression of NAFTc1, c-fos and Mitf. Blue colored arrow-ended lines: stimulation/activation; Black circle-ended dash lines: inhibition. Red colored pointed arrows: transcriptional activation; Black colored closed circle: transcriptional repression.

Mechanistically, these actions in HSCs are mediated through the differential regulation of the downstream transcription factor Maf basic leucine zipper (*bZIP*) transcription factor B (MafB) [33,34,65]. MafB is a negative regulator of osteoclast differentiation and is a direct target gene of C/EBPβ-LAP [33,34,65]. MafB represses the expression of several osteoclast transcription factors including NFATc1, c-fos and microphthalmia-associated transcription factor (Mitf) [33,65] and thereby, inhibits osteoclast differentiation (Figure 3). Consistent with this, it has been reported that repression of MafB resulting from either targeted deletion of LAP or forced expression of LIP was sufficient to induce osteoclast differentiation in HSCs [34]. Taken together, these studies indicate that the ratio of the transactivator to repressor isoforms of C/EBPβ is a key determinant of HSC fate by linking the mTOR pathway to the osteoclast differentiation program through regulation of MafB expression (Figure 3).

## 4. Bone Marrow Adipose Tissue (BMAT)-Secreted Molecules Regulating Osteoclast Differentiation

Accumulating evidence supports a major role for BMAT-secreted molecules in influencing the osteoclast differentiation program [6,36,37,46,66,67]. This is likely to be tissue-specific (to BMAT) since the major osteoclastogenic signal RANKL is reported to be highly expressed in mature bone marrow adipocytes but not in mature adipocytes at other sites such as peripheral fat depots [46]. This was demonstrated most convincingly with conditional deletion of a floxed parathyroid hormone/parathyroid hormone-related peptide receptor (PTH1R) gene through bone marrow MSC-specific Cre recombinase expression driven by paired-class homeobox transcription factor 1 (Prx1) [46]. The selective ablation of MSC PTH1R signaling resulted in increased BMAT, increased osteoclast differentiation and bone resorption as well as a low bone mass phenotype [46]. Interestingly, BMAT was determined to be the source of the elevated RANKL linked to increased bone resorption in this mouse model suggesting a major role for bone marrow adipocytes in promoting bone resorption during disease conditions [46]. These studies further suggest that in addition to the interplay of adipogenic transcription factors with the osteoclastogenic program, bone marrow adipocytes are a major source of RANKL that drives osteoclast differentiation and bone resorption under disease conditions. 

In addition to RANKL, adipokines such as chemerin, resistin, visfatin, leptin, adiponectin and omentin-1, lipids and fatty acids have been shown to influence the osteoclast differentiation program. For example, our previous studies reported that chemerin [66,67] signals through the cognate receptor CMKLR1 in HSCs to induce their differentiation into mature osteoclasts [36]. Chemerin/CMKLR1 signaling was found to regulate expression of the key osteoclast transcription factor NFATc1 and thereby, induce osteoclast differentiation and matrix resorption [36]. An experimental approach using chemerin neutralization identified a near complete blockade of osteoclast differentiation in HSCs suggesting that a basal level of chemerin is required for HSC osteoclastogenesis [36]. Moreover, it is noteworthy that the chemerin neutralization approach also resulted in a blockade of adipocyte differentiation of bone marrow MSCs [66], and thereby could exert a positive influence on the osteoblastogenic program of MSCs [23,68]. Therefore, approaches to block chemerin signaling or neutralize the chemerin protein in patients’ bone marrow may have therapeutic value for treating disorders of bone loss due to combined benefits resulting from inhibition of bone resorption due to blockade of HSC osteoclastogenesis and enhanced bone formation due to stimulation of MSC osteoblastogenesis. Similar to chemerin, resistin has been reported to exhibit a positive influence on the osteoclast development through a mechanism involving activation of nuclear factor of kappa B (NFkB) signaling [37]. The adipokine visfatin, also known as nicotinamide phosphoribosyltransferase (Nampt) or pre-B-cell colony-enhancing factor is essential for the biosynthesis of the coenzyme nicotinamide adenine dinucleotide (NAD) [69]. In contrast to chemerin, some reports suggest an inhibitory role for visfatin in the osteoclast differentiation program [69,70]. For example, Baek et al. (2017) reported that visfatin inhibited the phosphorylation of various early signal transducers, including c-Jun N-terminal kinase, Akt, glycogen synthase kinase-3 β, Bruton’s tyrosine kinase and phospholipase C γ-2 to suppress RANKL-induced osteoclastogenesis [70]. However, other studies using FK866, an inhibitor of visfatin, have demonstrated that visfatin is also required for the recruitment of osteoclasts to the bone surface [66]. Similar to visfatin, other adipokines such as leptin, adiponectin and omentin-1 also negatively impact bone resorption by affecting the osteoclast differentiation program [6,7,71]. In addition to direct actions on HSCs, adipokines such as leptin and omentin-1 can also modulate osteoclast differentiation indirectly by acting on MSCs to alter their production of RANKL and osteoprotegerin (OPG), a neutralizer of RANKL, thereby modifying the net stimulus for osteoclastogenesis in HSCs [6,72,73,74].

## 5. Future Perspective for Biomarkers and Therapeutic Targets

A primary goal in the field of musculoskeletal research is to identify reliable therapeutic targets to prevent the onset and progression of skeletal pathologies associated with bone loss. There is currently no validated biomarker for predicting the early stages of bone-loss disorders. Several promising candidates have been investigated including carboxy-terminal crosslinking telopeptide of type I collagen, a bone resorption marker, and procollagen type I N propeptide, a marker for bone formation in osteoporotic serum [75]. Serum levels of osteoclast-derived protein cathepsin K have also been examined as a candidate biomarker [75]. However, several physiological conditions and many disease states can modify the levels of bone turnover markers and thus, render them neither fully specific nor sufficiently sensitive for their use as an early stage marker [76]. In addition, there is presently no reliable candidate for the application of biomarkers to map treatment effects. In order to improve therapeutic outcomes, the identification of potential biomarkers and the underlying mechanisms are urgently required. Recently, bone marrow fat analysis has attracted attention as a diagnostic test for the early diagnosis of chronic diseases such as osteoporosis based upon the generally accepted inverse relationship between bone marrow fat and bone health. Consistent with this, some studies have successfully used bone marrow fat analyses as a novel imaging biomarker in the clinical diagnosis of postmenopausal fragility fractures [77]. Both the amount of and nature (level of fat unsaturation) of bone marrow fat were found to reliably identify differences between fracture and nonfracture patients [77]. Although promising, analyses using such imaging techniques are limited to a single skeletal region (commonly a segment of the lumbar spine) that may not represent the state of disease in the entire skeleton [77]. Since both vertebral and peripheral fractures are common after menopause transition or with diabetes mellitus, serum biomarkers may prove to provide a more reliable representation of the entire skeleton than the narrow analyses of a limited bone segment. In this regard, bone marrow adipose tissue-secreted molecules such as adipokines (including chemerin, visfatin, omentin, adiponectin and resistin), fatty acids, extracellular vesicles and other BMAT-derived cytokines such as RANKL are potential biomarkers for the diagnosis and treatment of osteoporosis and related disorders. However, the complex interplay between adipogenic and osteogenic factors, production of these molecules by both peripheral white adipose tissue and bone marrow adipocytes as well as the context-specific relationship to bone homeostasis, highlight the need for further investigation into the assessment of the clinical utility of adipokines as diagnostic markers and/or therapeutic targets for drug development. In this regard, some experimental and clinical studies have reported paradoxical results or inconsistent findings regarding the relationship between the bone marrow fat and bone loss diseases [78,79]. For example, some animal models such as the WBB6F1/J-KitW/W-v mice with loss-of-function mutations on c-kit gene [80] and 11beta-hydroxysteroid dehydrogenase type 1-knockout mice [81] exhibit bone loss without the expected increase in marrow adiposity. These contradictory findings suggest that the correlation between marrow adiposity and bone loss in diseases is context-dependent and varies depending upon the stimulus that drives the bone loss. Therefore, it is essential that these factors are considered in order to provide balanced insights into the mechanisms of osteoporosis and related bone loss disorders.

Clinical evidence shows that the currently available antiresorptives such as bisphosphonates and denosumab, and anabolics such as teriparatide, reduce vertebral, nonvertebral and hip fractures in postmenopausal women [82]. However, there is also clear clinical evidence of alarming adverse effects such as atypical subtrochanteric fractures, osteonecrosis of the jaw, increased risk for the development of cardiovascular diseases (atrial fibrillation and myocardial infarction), upper gastrointestinal problems, hypocalcemia, myalgia, cramp and limb pain with the use of antiresorptives [82]. Similarly, the use of teriparatide is associated with several untoward effects including upper gastrointestinal symptoms, hypercalcemia, renal side effects and hypercalciuria [82]. Moreover, the primary therapeutic benefit of reduced fracture risk in high-risk patients requires long-term treatment even with the most commonly used first-line bisphosphonate drugs [82]. Thus, furthering our fundamental understanding of disease mechanisms is essential to develop superior therapeutic applications. In this regard, recent studies identified marrow adipose tissue accumulation as the primary factor driving the early steps of bone marrow niche dysfunction, impaired bone tissue renewal and repair [83]. Studies such as these have stimulated renewed interest in examining the link between marrow fat and bone loss and have highlighted this as an exciting area of research to identify novel approaches for osteoporosis disease management both from a diagnostic and therapeutic perspective. 

In addition to the relationship between bone loss and marrow fat during disease manifestation, similar evidence exists for a relationship for the treatment of osteoporosis with most of the currently available therapies including antiresorptives and anabolics. In this regard, a number of antiresorptives and anabolic drugs have been reported to increase bone mass in parallel with a reduction of bone marrow fat [84,85,86,87]. Indeed, bisphosphonates that reduce bone loss by specifically binding to hydroxyapatite of bone and thus interfering with osteoclast function were also reported to decrease the amount of marrow fat during the course of therapy [84,85]. These findings also suggest that osteoclastogenic pathways may also exert a positive influence on adipocyte programming. Similarly, anabolic agents that directly promote osteoblastogenesis, suppress osteoblast apoptosis and thereby enhance bone formation, are also reported to reduce marrow fat accumulation in patients undergoing therapy [86,87]. Notably, this suppression of adipogenesis was found to be restricted to the bone marrow adipose tissue without impacting other white adipose tissue depots [86,87]. This further supports the regulation of bone mass through the cellular triad of osteoclast/osteoblast/adipocyte interactions particular to the marrow microenvironment. Future strategies to identify mechanisms between these interconnections such as alterations in adipokine signaling and the levels of marrow fat-derived substances during disease progression and therapy, and approaches to target bone marrow adipogenic events to abrogate the abnormal osteoclast formation could be of value in the diagnosis and treatment of osteoporosis.
